# Parent and Clinician Perspectives on Diagnostic Testing for Children With Diarrhea: A Qualitative Study

**DOI:** 10.1001/jamanetworkopen.2025.31000

**Published:** 2025-09-09

**Authors:** Anna Jones, Aparna Mangadu, Sarah Dallas, Olivia Hanson, Katherine Arn, Per Gesteland, Julia E. Szymczak, Andrew T. Pavia, Daniel T. Leung, Melissa H. Watt

**Affiliations:** 1 Department of Pediatrics, University of Utah School of Medicine, Salt Lake City; 2 Division of Infectious Disease, Department of Internal Medicine, University of Utah School of Medicine, Salt Lake City; 3 Intermountain Health, Salt Lake City, Utah; 4 Division of Epidemiology, Department of Internal Medicine, University of Utah School of Medicine, Salt Lake City; 5 Division of Pediatric Infectious Disease, Department of Pediatrics, University of Utah School of Medicine, Salt Lake City; 6 Department of Population Health Sciences, University of Utah School of Medicine, Salt Lake City

## Abstract

**Importance:**

Advances in diagnostics have enabled the detection of more gastrointestinal pathogens, but misuse of diagnostics can lead to inappropriate antibiotic use and excess financial burdens. Ensuring appropriate use of diagnostics is crucial for optimizing patient care and promoting stewardship of health care resources.

**Objective:**

To elicit parents’ and clinicians’ perspectives on expectations for care of pediatric diarrhea with a focus on diagnostic testing and to evaluate the potential for an electronic clinical decision support tool (ECDST) to improve appropriate use of diagnostics.

**Design, Setting, and Participants:**

In this qualitative study, purposeful sampling was used to recruit parents of children who had sought care for diarrhea and clinicians who care for children with diarrhea from 5 urgent care sites and 1 emergency department in Utah. Recruitment ended after achievement of inductive thematic saturation. Participants were interviewed between June 15, 2023, and January 24, 2025.

**Main Outcome and Measures:**

Key themes on expectations for management of pediatric diarrhea and perceptions of an ECDST. Interviews elicited expectations for diagnostic testing and treatment of pediatric diarrhea as well as the perceived utility of an ECDST. Analysis followed an applied thematic analysis framework.

**Results:**

Forty-four parents (40 [91%] female; median age, 34 years [range, 21-47 years]) and 16 clinicians (11 [69%] female; median age, 42 years [range, 29-59 years]) were interviewed. Three motivators were identified for parents to seek clinical care for a child with diarrhea: (1) reassurance, (2) understanding the cause of symptoms, and (3) appropriate treatment. Parents viewed diagnostic testing as a tool for meeting these expectations. Clinicians expressed skepticism for the value of diagnostics in informing etiology or treatment but reported that they may order diagnostics to reassure parents. Many clinicians acknowledged the benefits of an ECDST to assist with evidence-based decision-making for diagnostics and facilitate communication with families.

**Conclusions and Relevance:**

This qualitative study identified both similarities and tensions between parents and clinicians in expectations for pediatric diarrhea care, especially pertaining to the role of diagnostics. The findings suggest that strategies such as the use of ECDSTs are needed to resolve tension in care expectations, facilitate diagnostic stewardship, and optimize care for pediatric diarrhea.

## Key Points

**Question:**

What are the expectations of parents and clinicians when managing pediatric diarrhea?

**Findings:**

In this qualitative study of 44 parents and 16 clinicians, parents identified 3 motivators for seeking clinical care for a child with diarrhea: reassurance, understanding the cause of symptoms, and appropriate treatment. Clinicians expressed skepticism for the value of diagnostics in informing etiology or treatment but reported they may order diagnostic testing to reassure parents.

**Meaning:**

The findings suggest strategies are needed to resolve tension between clinicians and parents in care expectations, facilitate diagnostic stewardship, and optimize care of children with diarrhea.

## Teaser

This qualitative study examines perspectives of parents and clinicians about expectations for care of children with diarrhea as well as diagnostic testing for pediatric diarrhea.

## Introduction

Diarrheal illness, primarily acute infectious diarrhea, remains a leading cause of morbidity and mortality among young children worldwide, with an estimated 1.7 billion cases and approximately 450 000 deaths per year.^1,2^ In the US, infectious gastroenteritis continues to place a substantial burden on the health care system.^3,4^ The recent development of multiplex polymerase chain reaction (PCR) panels for gastroenteritis has enabled the rapid identification of multiple gastrointestinal pathogens (up to 22 bacteria, viruses, and parasitic organisms) from stool samples.^5,6^ Multiplex molecular methods have high sensitivity and specificity for gastrointestinal pathogens and have a faster turnaround time than traditional culture-based methods.^6,7^ However, differentiation between a disease-causing pathogen and asymptomatic carriage can be challenging, especially when multiple pathogens are detected.^8,9^ Although the Infectious Diseases Society of America 2017 clinical practice guidelines for the diagnosis and management of infectious diarrhea provide broad recommendations for when diarrhea-related diagnostics should be used, clear guidelines specific to the use of multiplex PCR panels do not exist.^8,10^ When used appropriately, multiplex PCR panels can facilitate the prompt initiation of organism-specific treatment for some bacterial and parasitic infections and alleviate the need for more invasive workups and repeat health care encounters.^11,12^ However, especially given the self-limited nature of most cases of pediatric gastroenteritis, the misuse of these panels can lead to inappropriate use of antibiotics, increased financial burden within the health care system, and potential time and financial costs for families.^13,14,15^

Overuse of diagnostic testing for pediatric gastroenteritis can raise health care costs without improving patient management.^14^ Conversely, underuse can result in inappropriate treatment and failure to detect outbreaks and can increase parental anxiety. Drivers of overuse may include a desire to meet patient expectations and achieve patient satisfaction or to diagnose illness without suggestive symptoms.^16,17^ Failure to use diagnostic tests when appropriate may be driven by lack of clear guidelines or lack of knowledge.^18^ Diagnostic stewardship, or optimizing the appropriate use of diagnostic tests to improve the diagnostic process and ultimately treatment, is crucial to promoting stewardship of health care resources and optimizing patient care.^19^

Clinical decision support tools (CDSTs) are designed to assist clinicians in making informed and accurate diagnostic and prognostic decisions using available characteristics of the patient and the larger context.^20^ These tools can improve health care process outcomes, including prescribing and diagnostic tool use patterns.^21^ A CDST should be designed based on the insights of end users (eg, clinicians who treat children with diarrheal illness as well as the parents or guardians of these children), as these tools are often used to facilitate shared decision-making with families.^22,23,24^ CDSTs currently support diagnostic decision-making in other pediatric conditions, such as imaging for pediatric head trauma, and prior studies have evaluated CDSTs in adult populations for decision-making with multiplex PCR panels for gastroenteritis.^25,26^ To better understand current decisions about diagnostic testing and the potential for a CDST to assist in diagnostic decision-making for pediatric diarrhea, this study aimed to identify parents’ and clinicians’ expectations for care of children with diarrhea, with a specific look at diagnostic testing and attitudes toward using a CDST.

## Methods

### Study Design

In this qualitative study, we conducted individual in-depth interviews (IDIs) with parents or guardians of children who had been evaluated for a primary complaint of diarrhea and clinicians working at urgent care and emergency department (ED) facilities who provide care for children with diarrhea. The interviews evaluated participants’ perspectives on and expectations for receiving care for a child with diarrhea or treating a patient with pediatric diarrhea as well as the potential use of an electronic CDST (ECDST) to guide diagnostic clinical decision-making for pediatric diarrhea. This study was approved by the University of Utah institutional review board. Participants provided written informed consent. Reporting of this study follows the Consolidated Criteria for Reporting Qualitative Research (COREQ) guideline.^27^

### Sample and Recruitment

We recruited participants via purposeful sampling; recruitment ended once inductive thematic saturation was reached.^28,29^ Parents or guardians were recruited from 5 academic urgent care sites in the Salt Lake City, Utah, metropolitan area. Clinicians were recruited from the 5 urgent care sites as well as 1 pediatric ED. Research assistants (A.M., S.D., O.H.) identified eligible parents or guardians by reviewing visit logs and evaluating clinical encounters in which the patient was under 6 years of age with a primary complaint of diarrhea and the parent’s or guardian’s primary language was English or Spanish (eAppendix 1 in Supplement 1 provides the research team description). Eligible parents or guardians were recruited using email and short message service (SMS) outreach. Recruitment alternated between email and SMS for a maximum of 3 attempts or until a response was received, whichever came first.

Eligible clinicians were practitioners at the same facilities who had prescribing authority (eg, physicians, nurse practitioners, and physician associates) and treated children with diarrheal illness. Study staff recruited clinicians via email outreach.

### Data Collection

The IDIs were conducted via videoconference for clinicians or via videoconference or telephone for parents or guardians, depending on participant preference, between June 15, 2023, and January 24, 2025. No repeat interviews were required, and no nonparticipants were present during the interviews. The parent or guardian interview guide (eAppendix 2 in Supplement 1) included open-ended questions on expectations when receiving care (including specific questions on diagnostics and treatment) for pediatric diarrheal illness at health care facilities, understanding and knowledge about antimicrobial stewardship, opinions on their personal role in antimicrobial stewardship, knowledge about drawbacks of antibiotics, and perspectives on the utility of an ECDST for pediatric diarrhea. The clinician interview guide (eAppendix 3 in Supplement 1) included open-ended questions about clinicians’ management of pediatric diarrhea, including their use of diagnostics and antibiotics, perceptions of parents’ expectations, perspectives on diagnostic and antimicrobial stewardship, and opinions about the utility and feasibility of an ECDST for pediatric diarrhea. Demographic information, including age, gender, and participant-identified race and ethnicity, was collected during the interview to ensure representation within the study population. Race and ethnicity categories were Asian; Black or African American; Hispanic or Latino; White, not Hispanic or Latino; and unknown or not disclosed.

Prior to conducting the IDIs, each participant signed a digitally delivered consent form. The IDIs were audio-recorded, and the recordings were transcribed verbatim. At the conclusion of the interview, participants were mailed a gift card as compensation.

### Statistical Analysis

Analysis of the IDIs followed an applied thematic analysis framework^30^ using NVivo, version 14 (Lumivero).^31^ Data analysis was conducted concurrently with data collection. Stages of analysis included coding of the interview transcripts, analytic memo writing, and the identification of emergent themes across 3 domains: (1) parent motivations for seeking care for pediatric diarrhea, (2) clinicians’ management of parents’ expectations with regard to diagnostics, and (3) potential for an ECDST to manage diagnostic decision-making. Research team members (A.J., A.M., S.D., O.H., M.H.W.) developed a preliminary set of a priori deductive codes using the central topics of the questions in the interview guide (eAppendixes 2 and 3 in Supplement 1). Multiple team members (A.J., A.M., S.D., O.H.) iteratively coded the interviews and reached consensus on inductive codes based on emergent patterns. After coding, 4 team members (A.J., A.M., D.T.L., M.H.W.) reviewed code reports and wrote analytic memos, synthesizing codes and text excerpts to develop themes. Frequent meetings were held to discuss codes and themes, ensuring consensus on accurate data representation. The 4 team members (A.J., A.M., D.T.L., M.H.W.) agreed on inductive thematic saturation when no further emerging themes were identified, indicating data saturation.^32^

## Results

The sample of parents or guardians included 44 individuals who self-identified as the parent of a child seeking care for diarrhea. Forty parents (91%) were female and 4 (9%) were male, and median age was 34 years (range, 21-47 years). One parent (2%) identified as Asian; 2 (5%) as Black or African American; 15 (34%) as Hispanic or Latino; 22 (50%) as White, not Hispanic or Latino; and 4 (9%) had unknown or undisclosed race and ethnicity. Most parents spoke English as their primary language (40 [91%]) (Table). The sample of clinicians (n = 16) included 10 physicians (62%) and 6 nurse practitioners or physician associates (38%) with experience working at the 6 different health care locations. Eleven of the clinicians (69%) were female and 5 (31%) were male, with median age of 42 years (range, 29-59 years). Fourteen clinicians (88%) self-identified as White, not Hispanic or Latino and 2 (13%) had unknown or undisclosed race and ethnicity. The clinicians reported a median of 7 years (range, 2-25 years) of practice experience (Table).

**Table.** Demographic Characteristics of Clinicians and Parents

Characteristic	Participants^a^	
	Clinicians (n = 16)	Parents (n = 44)
Gender		
Female	11 (69)	40 (91)
Male	5 (31)	4 (9)
Age, median (range), y	42 (29-59)	34 (21-47)
Child age, median (range), mo	NA	21 (4-70)
Primary language		
English	NA	40 (91)
Spanish	NA	4 (9)
Race and ethnicity		
Asian	NA	1 (2)
Black or African American	NA	2 (5)
Hispanic or Latino	NA	15 (34)
White, not Hispanic or Latino	14 (88)	22 (50)
Unknown or did not disclose	2 (13)	4 (9)
Role		
Nurse practitioner	4 (25)	NA
Physician associate	2 (13)	NA
Physician	10 (63)	NA
Practice location or location visited		
Urgent care	8 (50)	44 (100)
Emergency department	8 (50)	NA
Years of practice, median (range)	7 (2-25)	NA

Abbreviation: NA, not applicable.

^a^ Data are presented as number (percentage) of participants unless otherwise indicated. Percentages may not sum to 100 due to rounding.

Interviews with parents occurred between 3 and 67 days (median, 13 days) after the clinical encounter for diarrhea. The following sections describe parents’ and clinicians’ expectations for care of children with diarrhea, with a specific look at diagnostic testing and attitudes toward using a CDST. The Figure provides a summary of the study findings.

**Figure.**
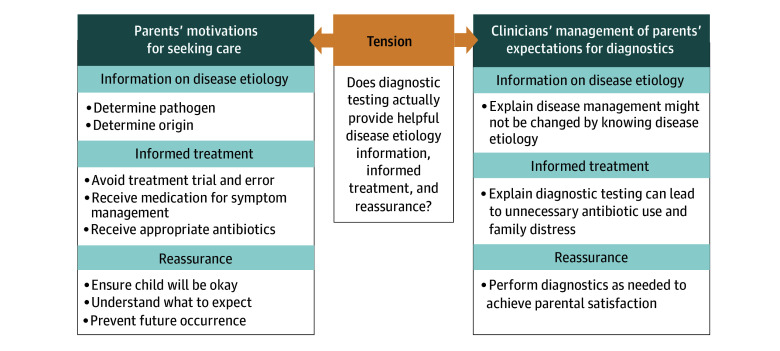
Summary of Study Findings

### Parent Motivations for Seeking Care for Pediatric Diarrhea

Three themes emerged as motivators for parents to seek clinical care for a child with diarrhea. These included (1) reassurance, (2) understanding the etiology, and (3) obtaining appropriate treatment.

#### Reassurance

When asked about their expectations for care, parents frequently mentioned the concept of reassurance, or “a little peace of mind” (mother of a child aged >3 years). For some parents, reassurance included validation of their current care methods:

I think we were just looking for them [clinicians] to take a knowledgeable look at him and just make sure that he’s doing okay. And let us know if we should be doing anything different. (Father of a child aged 1-3 years)

For other parents, reassurance meant feeling confident that their child’s condition would not deteriorate further or that they were not missing a serious underlying cause of their child’s symptoms:

I just want to make sure that...there’s not a bigger scarier thing that I’m missing, because I just don’t have the education to know how to detect it. (Mother of a child aged 1-3 years)

#### Understanding the Etiology

Understanding the etiology of the diarrheal illness was a priority for most parents. Many believed that diagnostic testing to identify the specific etiology of the illness would be useful. Parents indicated that knowing the etiology would offer desired reassurance and potentially inform treatment decisions:

I think a diagnostic test would bring more comfort, because you would know exactly what’s going on. And if it’s correct. If we wanted to just guess, I could Google it. (Father of a child aged 1-3 years)

Some parents expressed concern that without a diagnostic test, clinicians were merely speculating about the etiology and therefore may not have been using the most appropriate treatment:

I’d rather have a definitive test...Now what the procedure with the test is, I’m not sure. But I’d rather...have a test done just to pinpoint it and narrow down instead of just winging it. (Father of a child aged 1-3 years)

When a visit did not include diagnostic testing to assess the etiology of diarrhea, some parents expressed frustration:

I was hoping they would...do a test and kind of figure out what it was. But like, honestly, ...going there was pointless, because we got...no answers. (Mother of a child aged 1-3 years)

However, other parents were satisfied with a clinician’s knowledge and reassurance without further testing:

I think it’s okay if the doctor is just saying it’s a virus, like I don’t need a super definitive answer as long as they are sure. (Mother of a child aged >3 years)

#### Obtaining Appropriate Treatment

When seeking care for their child’s diarrheal illness, parents frequently reported a goal of symptom relief or “just ideas about how to treat [it] more effectively” (mother of a child aged 1-3 years):

Help me to give him...something or told me...if I have to do something to make him get better sooner. (Mother of a child aged 1-3 years)

Many parents confirmed their understanding that “antibiotics do not work for viruses” (mother of an infant aged <1 year). Most parents understood the difference between a viral and bacterial etiology of infectious diarrhea and did not expect antibiotics unless their child had a bacterial infection. Several parents also mentioned concerns about antibiotic resistance and adverse effects if using antibiotics inappropriately:

I know that bacterial would be if there’s a bacteria thing that really should get some help from antibiotics; viral usually just kind of has to run its course. And there’s not really a whole lot medication-wise, like antivirals don’t have as great of a selection, medication-wise, as antibacterial. (Mother of a child aged 1-3 years)

Parents generally believed that antibiotics would be appropriate if their child were confirmed to have a bacterial infection through a diagnostic test:

Because if he does have a bacterial infection that’s causing the diarrhea, then yes, I do want an antibiotic to help with the bacterial infection. (Mother of a child aged 1-3 years)

### Clinician Management of Parents’ Expectations With Regard to Diagnostics

Clinicians acknowledged that families want to understand the source of their child’s symptoms and that “information for some parents is really important” (ED physician with ≥10 years of practice). Multiple clinicians conveyed that parents were reassured and satisfied when diagnostic testing was performed.

[The] family feels like you’ve done more for them when you order a test. (Nurse practitioner, urgent care, <10 years of practice)

Some clinicians expressed that their perceived expectation that families want to have as much information as possible can influence their decision to order a diagnostic test:

Even if I don’t think that a GI [gastrointestinal] stool study is necessary, there are situations where...a family is not going to leave the [ED] happy without one. And so, I probably order them sometimes when they’re not truly indicated, but...I figured it’s not the worst thing in the world. (Physician, ED, <10 years of practice)

That said, clinicians thought that diagnostic testing for pediatric diarrhea was generally not warranted, except in unique cases (eg, bloody stools, prolonged duration of diarrhea, or travel history). Many clinicians stated that testing would not change their clinical management of patients:

The fact that it’s not going to give us information that we’re going to act on...if the kid’s well, there’s a good chance even if it is bacterial, they’ll get over it. (Physician, urgent care, <10 years of practice)

Some clinicians stated that further testing could even interfere with providing reassurance to families, as treatment decisions are not always straightforward using the multiplex PCR results:

The other con is trying to explain to family members that a positive result doesn’t necessarily mean we needed to do anything differently. (Physician, ED, ≥10 years of practice)

Beyond the perceived limited utility of diagnostic testing in managing pediatric diarrhea, clinicians expressed other considerations, including the substantial cost of the testing and the burden to families of collecting a valid stool sample:

I’m reluctant to get it if I really don’t think it’s gonna change my management, because it’s super expensive, and not all insurance covers it. (Physician, ED, ≥10 years of practice)

### Potential of an ECDST to Manage Diagnostic Decision-Making

#### Clinicians’ Perspectives

Clinicians had experience with ECDSTs for other disease processes and were interested in a tool that could help them make informed decisions about diagnostic testing for pediatric diarrheal illness. While clinicians typically reported that they were confident about when to order a diagnostic test, many expressed that an ECDST would bolster their confidence about whether diagnostic testing was warranted, especially in less straightforward scenarios:

The culture of clinical decision tools is really part of our culture as clinicians here. So, I think you would have people adopt that if it was available. (Physician, ED, ≥10 years of practice)

Multiple clinicians emphasized the importance of evidence-based decision-making and believed that a tool that incorporates “patient data, patient statistics” (nurse practitioner, urgent care, <10 years of practice) would support their clinical decision-making. An ECDST could also help clinicians to think more comprehensively about a clinical scenario:

It’d be helpful to have that algorithm pick up what we’re missing. And so, I think that’s a good thing to remind us. (Physician, ED, ≥10 years of practice)

Many clinicians also saw the benefits of using an ECDST to build trust and rapport with the patient’s family and provide them with reassurance that they are providing appropriate and evidence-based care:

If you’re willing to have that conversation with them in the room and...go through it with them, it just adds to that shared decision-making model. I think it adds trust...I think it does kind of back up our ability to defend why we’re doing what we’re doing. (Physician, ED, <10 years of practice)

A potential challenge of an ECDST that was shared by clinicians is that a decision tool could not be used in every pediatric diarrhea case, and understanding the appropriate application is important:

Any time there’s an algorithm, it doesn’t allow for...more subtleties. And I think with vomiting and diarrhea, especially vomiting, there’s...so many things that [it] can be that it’s hard to [account] for all of them in an algorithm. (Physician, ED, <10 years of practice)

#### Parents’ Perspectives

When we described the concept of an ECDST to parents, many had difficulty understanding. After additional explanation, parents were overall indifferent or indicated that they thought an ECDST would be useful and provide reassurance, especially if “evidence-based” (mother of a child aged 1-3 years):

So the more questionnaires, the more research, the better, in my opinion. So I do think that that would give me some peace of mind. (Mother of a child aged 1-3 years)

However, several parents expressed concerns that a tool does not account for nuances and would lead to “generalizing every kid” (father of a child aged 1-3 years), as opposed to providing patient-centered care. They also expressed concerns that a clinician would not use clinical reasoning if relying on a tool:

I think it could be good and could be bad...it could be bad because sometimes there is nuance...maybe the app says it’s not...applicable or not necessary, but in actuality, it is...because...the data is not infallible. (Mother of a child aged >3 years)

I don’t want them to make the app do all the work for them. I want them to also...think critically about...their own clinical training and experience and use that [to] help inform that decision as well. (Mother of a child aged 1-3 years)

## Discussion

In this study, we examined parent and clinician perspectives on management of pediatric diarrhea, with a specific focus on diagnostic testing. We synthesized participants’ similarities and tensions in expectations for care as well as their understanding of the role of a potential decision support tool (Figure). This study highlights drivers for diagnostic testing in children with diarrhea. Understanding these drivers is essential to develop solutions for inappropriate use of diagnostics and promote diagnostic stewarship.^19^ We identified 3 motivators for parents to seek clinical care for a child with diarrhea: (1) reassurance, (2) understanding the etiology, and (3) obtaining appropriate treatment. Together, these motivations prompted a desire for diagnostic testing. Clinicians’ perspectives on diagnostics suggested skepticism about diagnostics informing etiology or treatment, but clinicians reported a willingness to order diagnostic testing to reassure parents. An ECDST was perceived as a possible solution to mitigate this tension by serving as an objective tool. An ECDST has the potential to inform diagnostic testing decisions, although parents expressed some reservations due to fear of inappropriate generalization of their child’s clinical scenario.

The primary motivator for parents seeking care for a child with pediatric diarrhea was reassurance. For many parents, diagnostic testing was a means to gain reassurance about their child’s condition and prognosis. Diagnostic testing provides clinical data, which parents expect to be comforting. This concept has been reported in multiple medical contexts, including viral testing for upper respiratory infections in children as well as in annual wellness visits for adults.^33,34^ A recent study evaluating the clinical impact of multiplex PCR testing in pediatric diarrhea demonstrated that universal testing was associated with decreased rates of returning to care for the same illness, suggesting that knowing the etiology made parents and clinicians more comfortable with conservative management.^12^ However, universal testing was not associated with caregiver-reported confidence in their abilities to care for their child,^12^ which is in contrast to the notions expressed by caregivers in this study.

While clinicians don’t always agree with the need for diagnostic testing, they may acquiesce to diagnostic testing to appease parents’ desire for information and reassurance. This situation is not unique to pediatric diarrhea. A systematic review to understand the reasons behind overtesting across clinical settings found that interpersonal pressures, such as those from patients, are a growing issue in health care.^18^ For example, respiratory tests are often ordered to provide reassurance to families and clinicians.^33^ It is challenging for patients and families to appreciate the harms of diagnostic testing, and overtesting is traditionally a challenging concept for clinicians to communicate to patients.^35^ In this study, families expressed understanding of the harms of overtreatment, specifically the misuse of antibiotics, but they did not express concerns about potential harm of overtesting.

Advanced diagnostics allow more refined etiologic diagnoses and may improve treatment, but it is critical to know which patients would be likely to benefit. An ECDST may help clinicians identify where testing is the most useful for pediatric diarrhea and communicate this to families. An evidence-based tool could support a clinician’s conversation with families about the benefits and limitations of diagnostic testing and avoid unnecessary diagnostic testing simply for the purpose of reassurance, thus promoting diagnostic stewardship.^36^ Clinicians in this study expressed an interest in such a tool. Both clinicians and parents articulated the importance of evidence-based decision-making, and several existing CDSTs have been shown to increase adherence to evidence-based clinical practice guidelines for other pediatric conditions.^37,38^ However, parental concerns for loss of patient-centered care with such a tool highlights the importance of clinician communication when using algorithmic tools.

Several areas of future directions for ECDST development exist. This study’s findings suggest that in appropriate circumstances, an ECDST could help with diagnostic decision-making for children with diarrhea. Both parents and clinicians emphasized the importance of incorporating data based on relevant research into the tool. An ECDST for pediatric diarrhea should also provide clear guidance on inclusion criteria for application of the tool to facilitate safe and appropriate use. Additional studies are needed to assess clinician and parent satisfaction and clinical outcomes following implementation of an ECDST to ensure that such a tool is practical and provides the relevant information to reassure parents.

### Limitations

This study has several limitations. Interviews were conducted in the Salt Lake City metropolitan area, which potentially limits the generalizability of the results to other regions. Additionally, all parents were recruited from urgent care facilities, which could also limit generalizability of results to parents who seek care in other settings. Another limitation was the participant selection of interview modality (ie, videoconference vs telephone), as this may have led to different levels of engagement among participants.

## Conclusions

This qualitative study identified both similarities and tensions in parents’ and clinicians’ expectations for pediatric diarrhea care, especially as it relates to the role of diagnostics. The findings suggest that a CDST has the potential to support diagnostic stewardship by facilitating evidence-based clinical decision-making on diagnostic testing for children with diarrhea and could help clinicians give reassurance to families.

## Notes

Supplement 1. **eAppendix 1.** SRQR S6 Researcher Characteristics and Reflexivity **eAppendix 2.** Interview Guide for Patient Caregivers **eAppendix 3.** Interview Guide for Clinicians

Supplement 2.
Data Sharing Statement
