# Supplementary material for: Parent and Clinician Perspectives on Diagnostic Testing for Children With Diarrhea: A Qualitative Study
Source: JAMA Netw Open. 2025 Sep 9;8(9):e2531000. doi: 10.1001/jamanetworkopen.2025.31000 (PMC12421345; doi:10.1001/jamanetworkopen.2025.31000)
Supplement: Supplement 1. — eAppendix 1. SRQR S6 Researcher Characteristics and Reflexivity eAppendix 2. Interview Guide for Patient Caregivers eAppendix 3. Interview Guide for Clinicians [file jamanetwopen-e2531000-s001.pdf]

Supplementary Online Content

Jones A, Mangadu A, Dallas S, et al. Parent and clinician perspectives on diagnostic testing for children with diarrhea: a qualitative study. *JAMA Netw Open*. 2025;8(9):e2531000. doi:10.1001/jamanetworkopen.2025.31000

eAppendix 1. SRQR S6 Researcher Characteristics and Reflexivity

eAppendix 2. Interview Guide for Patient Caregivers

eAppendix 3. Interview Guide for Clinicians

This supplementary material has been provided by the authors to give readers additional information about their work.

© 2025 Jones A et al. *JAMA Network Open*.

eAppendix 1. SRQR S6 researcher characteristics and reflexivity.

Interviews were conducted by OH, AM, and SD who were research assistants at the time of the interviews. Author AJ is a pediatric resident and works in the Emergency Department where providers and some patients were recruited from but was not involved with recruitment or interviewing of providers or caregivers; additionally, when analyzing the interviews AJ was blinded to the specific provider or caregiver who provided the response. Senior author MHW, as well as JES had prior experience with qualitative research methods. Author ATP is an attending pediatric infectious disease physician and senior author DTL is an attending adult infectious disease physician. Author PG is a pediatric hospitalist. Authors ATP and PG work in the same institution as the Emergency Department providers in the study but were blinded to the names of the participants. Author KA was an adult infectious disease fellow at time of the study. Members of the team included both females and males.

© 2025 Jones A et al. *JAMA Network Open*.

eAppendix 2. Interview guide for patient caregivers

The purpose of our interview today is to get your feedback as a caregiver of a child who is presenting to health care with a primary complaint of diarrhea. We are mainly interested in understanding the expectations you have in getting the best care for your child.

As a reminder, your conversation with me today is for research purposes only. Nothing you say today will be shared with your doctor or any member of your clinical team. We hope you will feel comfortable talking openly and honestly about your experiences as a parent or caregiver. If any of my questions are not clear, please let me know, and I can repeat or reword the question.

Do you have any questions before we begin?

I. Introduction: experience with childhood diarrhea

First, I'd like to talk to you about the events leading up to coming to this clinic/hospital for your child's diarrhea.

1. Please tell me briefly why you decided to come to the clinic/hospital for your child's diarrhea.
    a. What were your main worries or concerns about your child's condition?
2. Prior to coming to this clinic/hospital, did you seek care anywhere else?
3. Prior to coming to this clinic/hospital, did you use any medications to help with your child's diarrhea? If YES, please describe)

Thank you for sharing your child's experience, and I'm so sorry about the recent illness. I want to step back for a minute and hear what you understand about pediatric diarrhea, especially the potential causes of diarrhea.

1. You said that diarrhea can happen because of an infection. Can you tell me what you understand about how that happens?
2. What do you know about the differences between illnesses caused by bacteria and viruses? Do those illnesses look different? Do you treat them differently?
3. What do you see as any benefits or risks of treating diarrhea using antibiotics?

Thank you for sharing that information with me. Now I want to return to your child, and when you brought them to the clinic/hospital.

II. Attitudes

Now I want to talk about what you expect when you bring your child with diarrhea to the clinic/hospital, and what is important to you.

1. When presenting to this clinic/hospital, what were you expecting to receive from the visit?
    a. In what ways did the visit meet your expectations?
    b. In what ways did the visit *not* meet your expectations?
2. For some families, it's very important for them to know exactly what is causing their child's diarrhea. How important is it for you to know this?
    a. Why do you say that this is important / not important?
    b. Do you need to have a test to determine the cause, or is it enough for the doctor to tell you what they think is causing the diarrhea?
3. For some families, it's very important for them to get medication (like antibiotic) for their child's diarrhea. How important is it for you to get treatment when your child has diarrhea?
    a. Why do you say that this is important / not important?
    b. [If important] What do you think that antibiotic do for your child?

III. Subjective norms

Thank you for telling me about your own expectations for how you want your child with diarrhea treated. Now I'd like to discuss how your friends and family think children with diarrhea should be treated.

1. In general, how do people in your community take care of a child when they have diarrhea?
    a. What signs and symptoms are most worrying / concerning?
    b. How do you decide what kind of care the child needs?
    c. How do you decide whether to give a child oral rehydration at home?
    d. How do you decide whether to withhold food / drink due to diarrhea?
2. How do you decide whether to take the child to the clinic?
    a. How do you decide where to seek care?
    b. Are there any times you might want to bring your child to the clinic, but don't?
    c. What are the barriers of taking the child to the clinic?

3. Who in your household makes the decision about how to treat a child with diarrhea, and when it is important to take the child to the clinic?

IV. Self-efficacy

Next I'd like to talk about how you talk with the nurses and doctors about your child's care when you come here.

1. How comfortable do you feel talking with your doctor about the treatment you would like for your child's diarrhea?
    a. What would make it more comfortable to talk to your doctor about your child's treatment?
2. How do you want to be involved in decisions about care for your child's diarrhea?
    a. What does being involved look like for you?

V. Behavioral intentions

Now I'd like to talk to you about the use of antibiotics for children with diarrhea.

1. In what circumstances do you think antibiotics are necessary when a child has diarrhea?

There is a lot of discussion these days about over-use or inappropriate use of antibiotics. By antibiotics, I mean drugs like ciprofloxacin, amoxicillin, azithromycin. The reason this is important is because if people use too many antibiotics, or don't use them correctly, then they can cause side effects, and in the long term, they can stop working.

1. Have you ever heard about this issue before? Tell me what you've heard.
2. As a caregiver for a sick child, what do you see as your personal role to reduce antibiotic use?
3. What drawbacks, if any, do you see of using antibiotics that are not clinically necessary?

VI. Utility and feasibility of eCDSTs

Our research team is in the process of developing an application on their phone or computer that providers would use to care for children with diarrhea. They would use the app would help them to make a decision about whether or not to order a diagnostic test, and whether or not to prescribe antibiotics.

1. How would you feel about your doctor or clinical using an application on their phone or computer to make a decision about whether or not they order a laboratory test to understand the origins of a case of pediatric diarrhea?
    a. Would this make you feel better or worse about the care your child receives? Why?
2. How would you feel about your doctor or clinical using an application on their phone or computer to make a decision about whether or not they prescribe an antibiotic for a case of pediatric diarrhea?
    a. Would this make you feel better or worse about the care your child receives? Why?
3. What information would you like the provider to share with you when using this application?

I have reached the end of my questions. Do you have anything to add on this topic?

© 2025 Jones A et al. *JAMA Network Open*.

eAppendix 3. Interview guide for clinicians

The purpose of our interview today is two-fold. First, we want to understand how health care providers like you manage complaints of pediatric diarrhea and vomiting. We are interested in both your use of diagnostic labs, and your use of treatments like antibiotics. Second, we want to get your feedback on the potential utility of a clinical decision support tool to help providers optimally manage cases of pediatric diarrhea and vomiting.

As covered in the consent form, our conversation today will be used for research purposes only. When we present the results, we will aggregate everything we hear so that no individual is identified. We hope you will feel comfortable talking openly and honestly about your experiences as a clinician. If any of my questions are not clear, please let me know and I can repeat or reword the question.

Do you have any questions before we begin?

I. Introduction: experience taking care of children with diarrhea and vomiting

First, I'd like to hear about your role as a clinician in this clinic/hospital, and your experience taking care of young children (under 5 years of age) who present with a complaint of diarrhea or vomiting.

1. Your clinical schedule may vary, but please estimate for an average month, how often do you treat children who have a complaint of diarrhea or vomiting?
    a. What setting do you care for these children
    b. How does the presentation and frequency vary by season?
2. Let's consider a previously healthy 2-year-old with 36 hours of vomiting and diarrhea. How would you treat this case? (probe: diagnostics, treatment)
    a. What features, if present, might change your approach?
    b. Do you make any specific recommendations for managing the diarrhea?
    c. What recommendations do you have for follow-up for the patient?

II. Behavioral intention

Now I'd like to talk to you about how you make clinical decisions when you see a child with diarrhea or vomiting. We are especially interested in decisions about diagnostics and decisions about antibiotic use.

First, let's talk about diagnostic work up. By this we mean doing a stool-based diagnostic test to help with the management of a case of diarrhea or vomiting.

1. How do you decide whether or not to order a stool-based diagnostic test for a child with diarrhea or vomiting? (or summarize from last answer: information about the patient, information about the broader environment)
    a. What pros and cons do you consider when deciding whether or not order a stool-based diagnostic test (GI PCR)?
    b. How does the cost of diagnostic tests influence your decision making? (Does it vary by patient?)
    c. How confident do you feel about your ability to make the best decision of whether or not to order a diagnostic lab?
        i. What information would make you feel more confident?
    d. Any other stool-based test (other than GI PCR) you order for children under 5 with diarrhea or vomiting? (if so, what circumstances)
2. How do you decide whether or not to prescribe an antibiotic for a child with diarrhea or vomiting? (information about the patient, information about the broader environment)
    a. What pros and cons do you consider when deciding whether or not to prescribe an antibiotic for a child with diarrhea or vomiting?
    b. How does the cost of antibiotics influence your decision making? (Does it vary by patient?)
    c. How confident do you feel about your ability to make the best decision of whether or not to prescribe an antibiotic?
        i. What information would make you feel more confident?
    d. What types of antibiotics would you consider prescribing?

III. Subjective norms (patients/caregivers)

Now I'd like to ask you about your conversations with the parents or caregivers of your patients, and how this informs your decisions about whether to order a stool-based diagnostic test or giving an antibiotic.

1. What do you think are most families' expectations when they present with a child with diarrhea or vomiting?
    a. In your opinion, how important is a diagnostic test for the family?
    b. In your opinion, how important is an antibiotic prescription for the family?

2. How do you communicate with families about their expectations?
3. How do families' expectations inform your practice?

V. Attitudes and Social Norms (clinical stakeholders)

Now, I'd like to talk to you about something called "diagnostic stewardship".

1. When you hear the term "diagnostic stewardship", what does this mean to you?
    a. How does this inform your clinical practice?
    b. Is there a culture of diagnostic stewardship at your clinical practice location? Please describe.
    c. Diagnostic stewardship refers to the appropriate use of laboratory testing to guide patient management, including treatment, in order to optimize clinical outcomes and limit the spread of antimicrobial resistance.
2. When you hear the term "antimicrobial stewardship", what does this mean to you?
    a. How does this inform your clinical practice?
    b. Is there a culture of antibiotic stewardship at your clinical practice location? Please describe.

VI. Utility and feasibility of eCDSTs

We are developing a clinical decision support tool that providers could use when a child with diarrhea or vomiting presents at an Urgent Care or ED. Based on the patient's clinical data and population/environmental trends, the tool would provide evidence-based decision support for both ordering diagnostic labs and also prescribing antibiotics.

1. What are your experiences with using clinical decision support tools in your current practice? (probe: example, pros and cons)
2. What do you think about a clinical decision support tool for pediatric diarrhea or vomiting?
3. What do you see as some of the challenges / drawbacks to using a clinical decision support tool when you treat children with diarrhea or vomiting?
4. What advice do you have in designing a clinical decision support tool to help providers manage pediatric diarrhea or vomiting?

I have reached this end of my questions. Do you have anything to add on this topic?

© 2025 Jones A et al. *JAMA Network Open*.
