# Supplementary material for: Parent and Clinician Perspectives on Diagnostic Testing for Children With Diarrhea: A Qualitative Study
Source: JAMA Netw Open. 2025 Sep 9;8(9):e2531000. doi: 10.1001/jamanetworkopen.2025.31000 (PMC12421345; doi:10.1001/jamanetworkopen.2025.31000)
Supplement: Supplement 2. — Data Sharing Statement [file jamanetwopen-e2531000-s002.pdf]

Data Sharing Statement

Jones. Parent and Clinician Perspectives on Diagnostic Testing for Children With Diarrhea. *JAMA Netw Open*. Published September 09, 2025. doi:10.1001/jamanetworkopen.2025.31000

Data
Data available: No

Additional Information
Explanation for why data not available: Data will not be made available in order to preserve anonymity of participants
